# Effects of Banana Resistant Starch on the Biochemical Indexes and Intestinal Flora of Obese Rats Induced by a High-Fat Diet and Their Correlation Analysis

**DOI:** 10.3389/fbioe.2021.575724

**Published:** 2021-01-27

**Authors:** Jinfeng Fu, Yuting Wang, Simin Tan, Juan Wang

**Affiliations:** School of Food Science and Engineering, South China University of Technology, Guangzhou, China

**Keywords:** banana resistant starch, biochemical index, gut microbiota, correlation analysis, obesity

## Abstract

The effects of banana resistant starch (BRS) on obesity-related metabolic and intestinal flora were investigated in a high-fat diet-induced obesity model. After 6 weeks of intervention, the glucolipid metabolism index [blood glucose (GLU), total cholesterol (TC), triacylglycerol (TG), low density lipoprotein-cholesterol (LDL-C), and high density lipoprotein-cholesterol (HDL-C)], hormone index [leptin (LEP), insulin (INS), ghrelin, adiponectin (ADP), and thyroxine (T4)], and 16S rRNA sequencing analyses were performed for each group to explore the regulating effect of intestinal flora and the mechanism of weight loss in obese rats. The results showed that (1) BRS intervention significantly reduced the levels of GLU, TG, TC, LDL-C, LEP, and INS (*p* < 0.01) and increased the contents of ghrelin (*p* < 0.05) and ADP (*p* < 0.01). (2) BRS could improve the diversity of intestinal flora and regulate the overall structure of intestinal microorganisms, mainly by upregulating the *Bacteroides*/*Firmicutes* ratio and the relative abundance of *Cyanobacteria* and downregulating the relative abundances of *Deferribacteres* and *Tenericutes* (at the phylum level). BRS could inhibit the proliferation of *Turicibacter*, *Romboutsia*, and *Oligella* and increase the abundances of *Bacteroides*, *Ruminococcaceae*, and *Lachnospiraceae* (at the genus level). (3) Some significant correlations were observed between the gut microbiota and biomarkers. *Turicibacter*, *Romboutsia*, and *Oligella* were positively correlated with GLU, TG, TC, LEP, and INS and negatively correlated with ghrelin and ADP. *Bacteroides*, *Parabacteroides*, and *Akkermansia* were negatively correlated with GLU, TG, and TC. Conclusion: BRS had promising effects on weight loss, which could be associated with the improvement in host metabolism by regulating intestinal flora.

## Introduction

Obesity (BMI ≥ 30 kg/m^2^) is a metabolic disease that results in weight gain due to the accumulation of a large amount of adipose tissue in the body (Prospective Studies Collaboration, 2009). The prevalence of obesity likely results from the interaction of multiple factors: heredity, environment, dietary intervention, physical activity, lifestyle, and so on. Sedentary lifestyle and a high-sugar high-fat diet appear to be the most important factors causing obesity (Chooi et al., 2019). With the development of society, the prevalence of obesity has risen dramatically year after year. In 2015, approximately 603 million adults and 107 million children were obese (Collaborators et al., 2017), which means that obesity has become a worldwide epidemic. Moreover, obesity is a prevalent manifestation of metabolic disorders, and accumulating evidence has demonstrated that TC, TG, LDL-C, INS, and LEP levels were significantly higher in obese than in normal weight people, while the concentrations of ADP and ghrelin decreased (Yildiz et al., 2004; Addante et al., 2011). Vekic’s work (Vekic et al., 2019), which focused on metabolic disorders in obesity, indicated that high concentrations of TG and LDL-C accompanied by decreased HDL-C concentrations are the main characteristics of dyslipidaemia. LEP and ADP are associated with inflammation since LEP stimulates adipose tissue to secrete inflammatory cytokines, while ADP acts as an anti-inflammatory adipokine. At the same time, INS has a higher concentration in plasma and can lead to insulin resistance, hyperglycaemia, and hyperinsulinaemia. In summary, obesity will increase the risk of a variety of diseases, such as cardiovascular diseases (hypertension, atherosclerosis, and hyperlipidaemia) (Lavie et al., 2014), type 2 diabetes (Malik et al., 2010) and some cancers (esophageal cancer, cholangiocarcinoma, and pancreatic cancer) (Nam, 2017), which cause great threats to global public health and have passive effects on the quality of human life and healthcare costs (Tremmel et al., 2017).

Approximately 1.5 kg of bacteria exists in our gut, and they are not only the densest but also the most diverse microbiome in the human body (Zhao, 2013). The intestinal flora is closely related to the health status of the host, and the composition of the gut microbiota varies due to the age of the host, living environment, dietary habits, and other factors (Delzenne and Cani, 2011). It plays a very important role in the physiological processes of the host, such as nutrient digestion, absorption, energy utilization and storage, and metabolism (Saad et al., 2016). Diet is considered to be a major factor affecting the structure of intestinal flora that transforms food ingredients into bioactive metabolites with different functions, which further regulate the composition of intestinal microorganisms and influence the host phenotype (Laparra and Sanz, 2010). A large number of studies have demonstrated that obesity is related to gut microbiota dysfunction and that dietary intervention has important impacts on intestinal flora to a certain extent, mainly manifesting as changes in gut microbiota structure and function, hindering the development of obesity (Portune et al., 2017).

Resistant starch (RS) is defined as the sum of the starch and products of starch degradation not absorbed in the small intestine of healthy individuals (Anonymous, 1991). RS is subdivided into five major types: RS_1_, RS_2_, RS_3_, RS_4_, and RS_5_ (Meenu and Xu, 2019). RS_1_ is found mainly in grains or seeds and is composed of a matrix of proteins that makes it difficult for enzymes to get close to the starch granules. RS_2_ is resistant to enzyme digestion and present in food such as raw potatoes and unripe bananas. RS_3_ is retrograded starch formed during the cooling of gelatinized starch in moist-heated food. RS_4_ is chemically modified starch due to crosslinking, esterification, and etherification. RS_5_ is formed by amylose with lipids, and the long carbon chains are the cause of RS_5_ resistance. As a new prebiotic (Sajilata et al., 2006), RS can prevent colon cancer, slow the release of glucose, and control weight gain and other physiological effects, and the fermentation of RS in the colon produces short-chain fatty acids (SCFAs), which improve the intestinal barrier environment and play a key role in the prevention and relief of metabolic syndrome.

Recent studies have pointed to the composition of intestinal flora in connection with RS. The higher levels of *Bifidobacterium*, *Akkermansia*, and *Allobaculum*, which were colonized by RS, could alleviate the development of obesity, and the proportions of *Bifidobacterium* and *Akkermansia* were positively correlated with gut weight and GLP-1 (Tachon et al., 2013). Furthermore, RS could stimulate a cluster of bacteria in the *Clostridia* class and increase the concentration of fecal butyrate to decrease the inflammatory response and improve insulin sensitivity (Sanchez-Tapia et al., 2020). In addition, *Bacteroides plebeius*, *Blautia producta*, and *Prevotella stercorea* were negatively associated with TC, while *Bacteroides ovatus*, *Bacteroides uniformis*, and *Bacteroides acidifaciens* were positively correlated with ADP, and all of them were enriched after RS intervention, although RS did not significantly enrich *Methanobrevibacter* spp. and *Eubacterium dolichum*, but they were correlated with weight and SCFA levels. *Methanobrevibacter* spp., *Ruminococcus gnavus*, and *Prevotella stercorea* were negatively correlated with LDL (Upadhyaya et al., 2016).

Banana resistant starch (BRS) belongs to the RS_2_ type and is the main ingredient of green banana, which comprises approximately 50% of unripe banana pulp (dry base). Our preceding study showed that BRS had a good effect on weight loss and improved the condition of the obese rats. After obese rats were treated with a dose of 2.5 g/kg BRS for 6 weeks, the body weight of rats in BRS group was significantly decreased by 9.06% (*p* < 0.05) and the fat accumulation was reduced, which especially decreased by 34.32% in the epididymis fat and 31.48% in the ratio of adipose tissues (including epididymis fat and renal fat) and weight (*p* < 0.05), compared with that of obese rats in model group. But there was no significant difference in food intake compared with obese rats. Histology analysis revealed that BRS alleviated hepatic steatosis and fatty liver in obese rats. All of these above will be reported in detail in another article.

There are some studies about food intervention to relieve obesity. Our preceding researches (Tan and Wang, 2018; Wang, 2018) report that BRS has a good effect on weight loss and improved the condition of the obese rats. Moreover, some literatures (Alvarado-Jasso et al., 2020; Wu et al., 2020) show that banana flour is benefit for reducing body fat. But few studies focused on the mechanistic role of the BRS-induced weight loss. Therefore, the anti-obesity mechanism of BRS mediating intestinal flora was studied in this article. It is reported that RS has many physiological benefits, including the management and control of glucose-metabolism related diseases such as type 2 diabetes and obesity (Niba, 2002). However, the microstructures of RS from different food sources are not same, so the physiological functions of RS may be different. Therefore, the health benefits of BRS could not be predicted without test. The structures and physicochemical properties of BRS differ from variety to variety (Wang et al., 2014, 2016). We found that some banana cultivar do not have good effects on weight loss (Tan and Wang, 2018). Thus, the cultivar of banana used here is proved with a positive influence on alleviating obesity by our previous studies.

The present study aimed to explore the effect of BRS on intestinal flora and discuss the correlation among intestinal flora, the glucolipid metabolism index and serum hormones in a high-fat diet-induced obesity model in order to understand the mechanism of BRS-induced weight loss. The significances of this study are as follows. Firstly, it could enrich the theoretical basic research related to RS. Secondly, it is a fundamental research about the functional ingredient of banana.

## Materials and Methods

### Materials

Banana resistant starch was provided by Natural Banana Healthy Food Co., Ltd., (Guangdong, China). Banana cultivar is *Musa ABB Dajiao*. Orlistat (approval number H20123131) was purchased from Zhien Pharmaceutical Co., Ltd., (Chongqing, China).

### Animals and Experimental Design

Male SD rats (100–110 g, 6 weeks old) were purchased from Guangdong Medical Laboratory Animal Center (GDMLAC, Guangdong, China) with the laboratory animal license number SCXK (Guangdong) 2013-0002. The normal chow diet (NCD: 55% nitrogen-free extract, 18% crude protein, 10% water, 8% ash, 4% crude fat, 5% crude fiber, 1.8% calcium, and 1.2% phosphorus, with total calorific value 327.6 Kcal/100 g) was obtained from Jiangsu Xietong Pharmaceutical Bioengineering Co., Ltd., (Jiangsu, China). A high-fat diet (HFD: containing 64% normal control diet, 15.0% lard, 15.0% sucrose, 5% casein, 0.6% calcium hydrogen phosphate, and 0.4% stone powder, with total calorific value 404.84 Kcal/100 g) was obtained from Guangdong Medical Laboratory Animal Center (GDMLAC, Guangdong, China).

**FIGURE 1:**
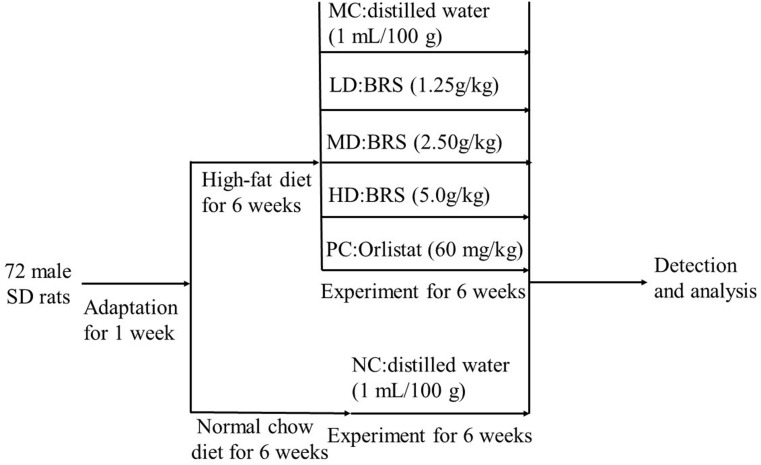
Animal experimental protocol and design. MC-model control group, LD-low dose group of BRS, MD-middle dose group of BRS, HD-high dose group of BRS, PC-positive control group, NC-normal control group.

All animals were raised in the specific pathogen-free (SPF) experimental animal room (constant temperature 21 ± 2°C, relative humidity 50 ± 10% and a 12-h light-dark cycle) of the Experimental Animal Center of South China Agricultural University with free access to water and food. The protocol and design of the animal experiment was showed in Figure 1. After 6 weeks of the experiment, all rats were fasted for 12 h and sacrificed with an intraperitoneal injection of 10% chloral hydrate. Blood samples were collected from the abdominal aorta and centrifuged at 3,000 r/min for 15 min at 4°C to obtain serum, which was stored at −80°C for measurements. The intestinal tract contents were obtained and stored at −80°C until analysis.

### Biochemical Assays

Total cholesterol (TC), total triacylglycerol (TG), low density lipoprotein-cholesterol (LDL-C), high density lipoprotein-cholesterol (HDL-C), and blood glucose (GLU) were determined by commercially available kits (Mindray Biomedical Electronics Co., Ltd., Shenzhen, China). Serum leptin (LEP), insulin (INS), adiponectin (ADP), thyroxine (T4), and ghrelin were measured using ELISA kits (ColorfulGene Biological Technology Co., Ltd., Wuhan, China). All measurements were carried out according to the manufacturer’s protocol.

### DNA Extraction and Sequencing

Total genomic DNA from large intestine samples (*n* = 3 per group) was extracted using the CTAB method, and the DNA concentration and purity were monitored on 1% agarose gels. Then, the V3–V4 hypervariable region of the microbiota 16S rRNA was amplified with the primers 341F (5′- CCTAYGGGRBGCASCAG-3′) and 806R (5′- GGACTACNNGGGTATCTAAT-3′). The PCRs consisted of the following program: initial denaturation at 98°C for 1 min, followed by 30 cycles of denaturation at 98°C for 10 s, annealing at 50°C for 30 s, elongation at 72°C for 30 s, and finally 72°C for 5 min. PCRs were carried out in 30-μL reactions with 15 μL of Phusion^®^ High-Fidelity PCR Master Mix (New England Biolabs), 0.2 μM of forward and reverse primers, and approximately 10 ng of template DNA. The same volume of 1 × loading buffer (containing SYBR green) was mixed with PCR products, and electrophoresis was performed on a 2% agarose gel for detection. Then, mixed PCR products were purified with a GeneJET^TM^ Gel Extraction Kit (Thermo Scientific, MA, United States). Purified amplicons were sequenced on an Ion S5^TM^ XL platform (Thermo Scientific, MA, United States), and 400/600 bp single-end reads were generated following the manufacturer’s recommendations by Nuohe Zhiyuan Technology Co., Ltd (Beijing, China).

### Bioinformatics Analysis

Cutadapt software (version V1.9.1) was used to filter and quality control the data to obtain raw reads, and then raw reads were detected and the chimera sequences were removed by usearch software (version v7.0.1090^1^) to obtain clean reads. Operational taxonomic units (OTUs) were performed by Uparse (version v7.0.1001^2^) with *a* ≥ 97% similarity threshold, and taxonomic analysis was conducted in comparison with the Silva database^3^ using the RDP classifier Mothur algorithm to annotate the taxonomic information of representative sequences for each OTU. The alpha diversity index and PCA analysis were calculated with QIIME (Version 1.7.0) and displayed with R software (Version 2.15.3). A heatmap was generated by using the vegan package in R software. Linear discriminant effect size analysis (LEfSe) was performed to characterize the differences among groups. The non-parametric factorial Kruskal-Wallis (KW) sum-rank test was used to evaluate species with significant differences in abundance between different groups, and linear discriminant analysis (LDA) was used to assess the magnitude of the impact of significantly different species. Spearman association analyses between gut microbiota and the metabolites and the *r*- and *p*-values were conducted using the MANTEL function. Moreover, the visualization work was done by the PHEATMAP function in the pheatmap package.

### Statistical Analysis

The data are shown as the mean ± SD. Statistical analysis was implemented using a one-way analysis of variance (ANOVA) followed by LSD *post hoc* test to determine the differences between groups. The results were considered significant when *p* < 0.05. Analyses were performed using IBM SPSS Statistics 24.0 (IBM, Chicago, IL, United States).

## Results

### BRS Ameliorated the Levels of Glucolipid Metabolism

The results showed that, compared with the NC group, the levels of GLU, TG, and TC markedly increased in the MC group (*p* < 0.01). The BRS treatment groups displayed a significant decrease in the levels of GLU (*p* < 0.01) and TG (*p* < 0.01) in comparison with the MC group, which gradually approached the NC group. The TC level in the LD (*p* < 0.01) and HD (*p* < 0.05) groups was extremely reduced, except for the MD group. In addition, an eventful reduction in LDL-C (*p* < 0.01) in the HD group, as well as a significant enrichment of HDL-C (*p* < 0.05) in the PC group, were observed compared with the MC group, but the HDL-C (*p* < 0.05) level in the HD group was lower than that in the MC group (Table 1). The above results showed that BRS could improve glucose and lipid metabolism abnormalities by lowering levels of TG and GLU. Effects of BRS on serum hormone levels.

**TABLE 1:** BRS improved glucose and lipid metabolism in obese rats (mmol/L).

**Groups**	**GLU**	**TG**	**TC**	**LDL-C**	**HDL-C**
NC	3.820.15^##^	0.440.07^##^	1.460.06^##^	0.460.09	0.700.08
MC	9.770.21**	0.650.03**	1.790.04**	0.480.05	0.640.05
LD	4.160.52^##^	0.440.05^##^	1.450.02^##^	0.490.10	0.610.10
MD	7.200.87^**##^	0.450.03^##^	1.780.09**	0.430.04	0.700.06
HD	3.780.3^##^	0.470.07^##^	1.610.07^#^	0.330.01^**##^	0.530.04^**#^
PC	6.760.56^**##^	0.570.03**	1.660.07^**#^	0.460.04	0.780.05^#^

The values were expressed as mean ± standard error (SD). Statistical analysis was performed using a one-way analysis of variance (ANOVA) followed by LSD *post hoc* test. *p < 0.05 and **p < 0.01 vs. NC; ^#^*p* < 0.05 and ^##^*p* < 0.01 vs. MC. “*”Means the difference was significant with NC at the 0.05 level.

To further explain the relationship between serum hormones and obesity, the levels of LEP, INS, ghrelin, ADP, and T4 were analyzed. As shown in Table 2, LEP and INS levels (*p* < 0.01) in the MC group significantly increased, while ADP (*p* < 0.01), ghrelin (*p* < 0.01), and T4 (*p* < 0.05) levels significantly decreased compared with the NC group. Compared with the MC group, the LEP and INS levels were crucially lower, while the ADP levels were sharply higher in the BRS and PC groups (*p* < 0.01). Additionally, the ghrelin levels (*p* < 0.05) of the HD group were obviously increased, but the LD and MD groups lacked a notable increase. The contents of T4 (*p* < 0.01) in the MD and HD groups were substantially enhanced compared with the MC group. It was interesting that the above serum hormones have a dose-effect relationship with BRS. It was implied that BRS were effective on reducing LEP and INS levels, increasing ghrelin and ADP levels, and then inhibiting obesity.

**TABLE 2:** BRS ameliorated the levels of serum hormones in obese rats.

**Groups**	**ADP (ng/mL)**	**INS (U/L)**	**LEP (ng/mL)**	**Ghrelin (mU/L)**	**T4 (ng/mL)**
NC	35.170.59^##^	8.460.19^##^	1.200.09^##^	1.050.11^##^	17.520.41^#^
MC	23.600.39**	15.150.40**	2.100.13**	0.760.03**	14.510.33*
LD	24.710.46^**##^	11.510.24^**##^	1.820.14^**##^	0.790.02**	14.730.72*
MD	28.160.41^**##^	11.181.73^##^	1.590.07^**##^	0.850.05**	17.910.67^##^
HD	34.440.68^##^	9.290.54^##^	1.370.09^**##^	0.920.06^*#^	18.661.27^##^
PC	26.180.51^**##^	9.230.23^##^	1.620.11^**##^	0.860.04**	16.271.18^#^

The values were expressed as mean ± standard error (SD). Statistical analysis was performed using a one-way analysis of variance (ANOVA) followed by LSD *post hoc* test. *p < 0.05 and **p < 0.01 vs. NC; ^#^*p* < 0.05 and ^##^*p* < 0.01 vs. MC.

### BRS Changed the Diversity of the Gut Bacterial Communities

Alpha diversity is used to assess the diversity of the microbial community in the sample, usually expressed as the alpha diversity index (Good’s coverage, Chao1, ACE, and Shannon) (Li et al., 2020). The Good’s coverage index reflects the depth and rationality of sample sequencing, which means that the closer the value is to 1, the lower is the probability of new OTUs in the sample. The Chao1 and ACE indexes were positively correlated with species richness. The Shannon index considers the uniformity of species distribution on the basis of richness; the higher the richness index and the uniformity, the stronger is the sample diversity. Therefore, the higher the Shannon index, the higher the biodiversity. The species diversity in different groups is shown in Table 3. The Good’s coverage of each group reached more than 0.99 (*p* > 0.05), indicating that each group had sufficient samples and that almost all of the sequences were detected. Compared to the NC group, the Chao1 and ACE indexes were not significantly different in the MC group (*p* > 0.05), but the Shannon index was significantly lower (*p* < 0.05). There was no difference in species richness in the intestinal flora of rats induced by the high-fat diet, but the highly uneven colony distribution led to a decrease in the community diversity of the MC group, suggesting that the high-fat diet caused an imbalance in the proportion of intestinal flora structure in rats. In the MD group, the Shannon index was significantly increased compared with that in the MC group (*p* < 0.05), reflecting that supplementation with BRS regulates intestinal microbial community diversity to a certain extent.

**TABLE 3:** Alpha diversity analysis of the gut microbiota in different groups.

**Groups**	**Good’s coverage**	**Chao1**	**ACE**	**Shannon**
NC	0.99900.0000	796.6124.63	793.7921.42	7.650.02^#^
MC	0.99900.0000	807.6920.23	808.0521.63	7.310.01*
LD	0.99900.0000	696.3716.12^*#^	711.4818.51^*#^	6.530.05^*#^
MD	0.99900.0000	781.7423.12	784.8820.20	7.430.07^*#^
HD	0.99900.0000	766.783.97	765.303.22^#^	7.280.03*
PC	0.99870.0005	826.0938.17*	821.5328.29	7.590.01^#^

The values were expressed as mean ± standard error (SD). Statistical analysis was performed using a one-way analysis of variance (ANOVA) followed by LSD *post hoc* test. *p < 0.05 vs. NC; ^#^*p* < 0.05 vs. MC.

**FIGURE 2:**
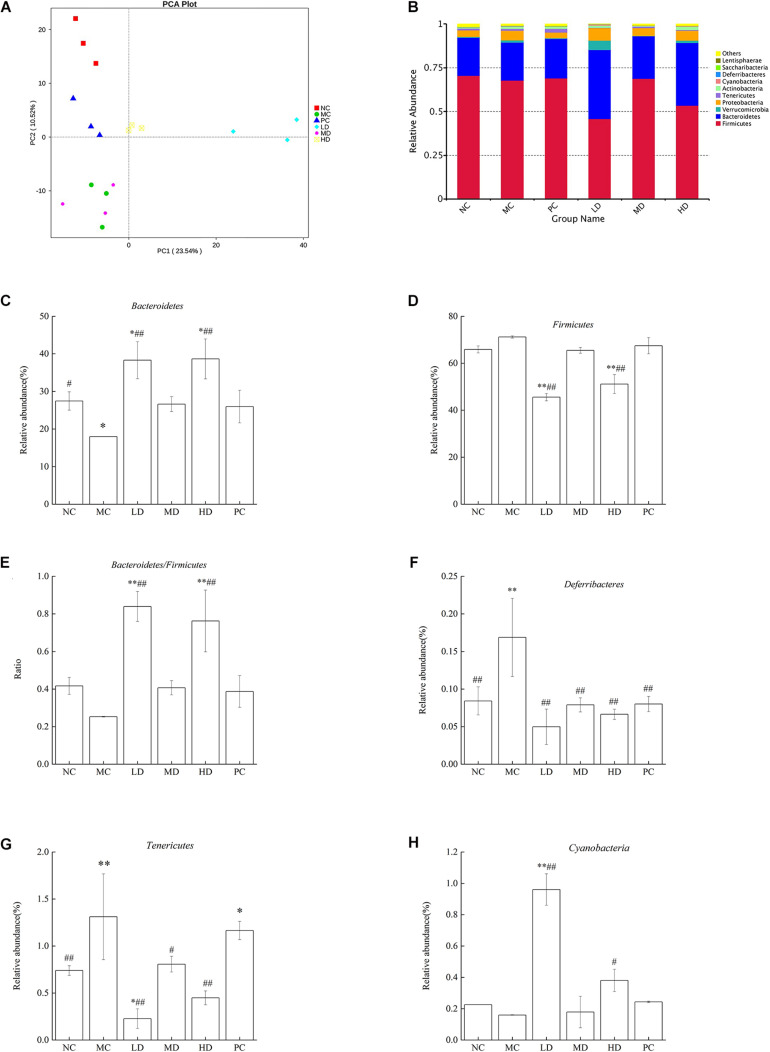
BRS alters the composition based on OTUs and the relative abundance of gut microbiota at the phylum level. **(A)** Principal component analysis (PCA) of gut microbiota based on OTUs. The abscissa represents the first principal component, the ordinate represents the second principal component, and the percentage represents the contribution of the principal component to the sample difference. Each point in the figure represents a sample, and samples from the same group are represented in the same color. **(B)** Bacterial taxonomic profiling at the phylum level of gut microbiota. **(C)** *Bacteroidetes*, **(D)** *Firmicutes*, **(E)** *Bacteroidetes/Firmicutes* ratio, **(F)** *Deferribacteres*, **(G)** *Tenericutes*, and **(H)** *Cyanobacteria*. Differences were based on ANOVA analysis followed by LSD *post hoc* test and denoted in graph bars as follows: ^∗^*p* < 0.05 and ^∗∗^*p* < 0.01 compared with NC; #*p* < 0.05 and ##*p* < 0.01 compared with MC.

Beta diversity is the analysis of microbial community structure of different samples to reveal the similarity of community composition between groups. Principal component analysis (PCA) is one of the methods used to evaluate the diversity of phylogenetic differences. In the PCA plot (Figure 2A), all groups exhibited an obviously distinct clustering of microbiota composition, suggesting that the samples in each group have a high degree of parallelism. The MC group was markedly separated from the NC group, with the LD, HD, and PC groups distributed in between, although the MD group overlapped with the MC group. The results implied that BRS intervention evidently altered the overall structure of the gut microbiota and improved disorders of the intestinal bacteria. It was observed that the diversity of intestinal flora was reduced significantly in obese rats. But the diversity could be recovered by BRS.

### Effects of BRS on the Composition and Structure of Intestinal Bacteria

To provide a better comprehension of the changes in the composition and structure of intestinal bacteria in response to BRS treatment, the bacterial species and abundance were determined at the phylum and genus levels among groups. In total, 12 phyla, 25 classes, 52 orders, 88 families, 192 genera, 78 species, and 1,043 OTUs were detected in this research. The study indicated that *Firmicutes*, *Bacteroidetes*, *Verrucomicrobia*, *Proteobacteria*, *Tenericutes*, *Actinobacteria*, *Cyanobacteria*, *Deferribacteres*, *Saccharibacteria*, and *Lentisphaerae* were phyla found in each group, and the bacterial composition was dominated by *Firmicutes* and *Bacteroidetes* (Figure 2B). Meanwhile, the relative abundance of different phyla in the gut microbes of each group was discussed for the purpose of accounting for the effect of BRS in intestinal bacteria. It was clear that the MC group fed a high-fat diet induced a major reduction in the relative abundance of *Bacteroidetes* (*p* < 0.05); however, although the difference was not significant, *Firmicutes* had a slight increase combined with the *Bacteroidetes*/*Firmicutes* ratio (B/F) and had a mild decrease (Figures 2C–E) in comparison with the NC group (*p* > 0.05), which was consistent with the literature (Dong et al., 2016). After 6 weeks of BRS treatment in obese rats, the LD and HD groups had a higher abundance of *Bacteroidetes* (38.31 ± 4.94%, 38.65 ± 5.33% vs. 17.98 ± 0.01%), a lower abundance of *Firmicutes* (45.55 ± 1.56%, 51.11 ± 4.03% vs. 71.18 ± 0.47%) and a higher B/F ratio (0.84 ± 0.08, 0.76 ± 0.16 vs. 0.25 ± 0.02) than the MC group (*p* < 0.01). As shown in Figures 2F–H, compared with the NC group, *Deferribacteres* and *Tenericutes* were significantly enhanced (*p* < 0.01), and *Cyanobacteria* was decreased in the MC group (*p* > 0.05), but the result did not achieve significance. Notably, BRS treatment had a beneficial effect on intestinal flora. The LD (*p* < 0.01) and HD (*p* < 0.05) groups had a considerable improvement in *Cyanobacteria* compared with the MC group (0.96 ± 0.10%, 0.38 ± 0.07% vs. 0.16 ± 0.02%); the LD (*p* < 0.01), MD (*p* < 0.05), and HD (*p* < 0.01) groups had a substantial reduction in *Tenericutes* compared with the MC group (0.23 ± 0.10%, 0.81 ± 0.08%, 0.45 ± 0.07% vs. 1.31 ± 0.46%); the *Deferribacteres* abundance of MC, LD, MD, HD, and PC was 0.17 ± 0.05%, 0.05 ± 0.02%, 0.08 ± 0.01%, 0.07 ± 0.01%, and 0.08 ± 0.01%, respectively, which were extremely lower with BRS treatment than that of the MC group (*p* < 0.01).

**FIGURE 3:**
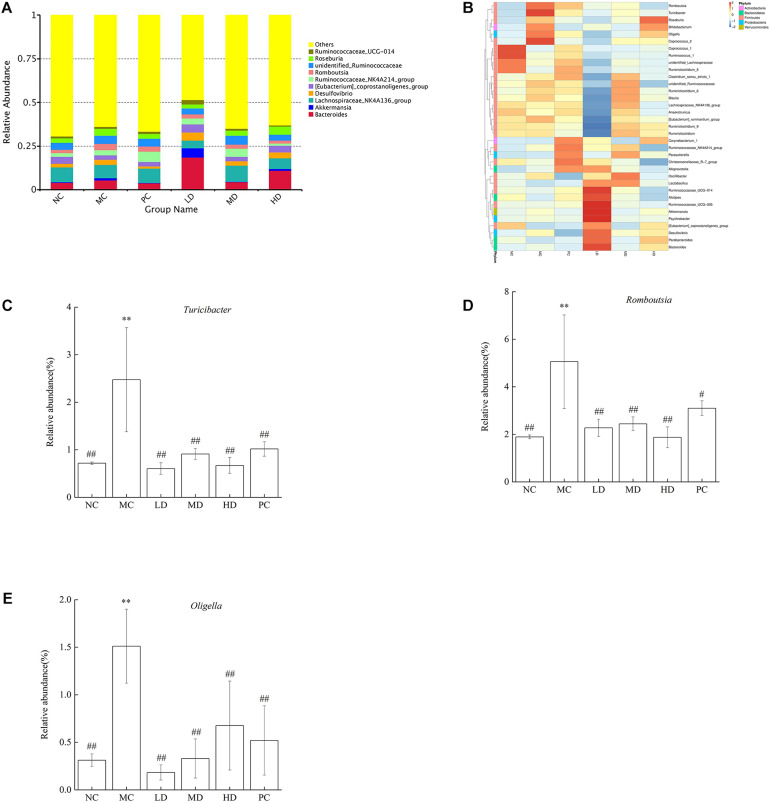
Relative abundance of gut microbiota at the genus level. **(A)** Bacterial taxonomic profiling at the genus level of gut microbiota. **(B)** Heatmap analysis at the genus level among different test groups displayed the changes in the relative abundance of identified genus. **(C)** *Turicibacter*, **(D)** *Romboutsia*, and **(E)** *Oligella*. Differences were based on ANOVA analysis followed by LSD *post hoc* test and denoted in graph bars as follows: ^∗^*p* < 0.05 and ^∗∗^*p* < 0.01 compared with NC; #*p* < 0.05 and ##*p* < 0.01 compared with MC.

The Top10 genera of relative abundance in different groups were *Bacteroides*, *Akkermansia*, *Lachnospiraceae_ NK4A136_group*, *Desulfovibrio*, *[Eubacterium]_ coprostanoligenes_group*, *Ruminococcaceae_NK4A214_ group*, *Romboutsia*, *unidentified_Ruminococcaceae*, *Roseburia*, and *Ruminococcaceae_UCG-014* (Figure 3A). In addition, the heatmap analysis showed that the MC group had more *Turicibacter*, *Romboutsia*, *Oligella*, *Roseburia*, *Coprococcus_2*, and *Bifidobacterium* than the NC group. As expected, BRS could regulate intestinal flora; the relative abundances of *Turicibacter*, *Romboutsia*, and *Oligella* were lower, and *Bacteroides*, *Parabacteroides*, *Desulfovibrio*, *[Eubacterium]_coprostanoligenes_group*, *Psychrobacter*, *Akkermansia*, *Ruminococcaceae_UCG-014*,*Ruminococcaceae_UCG-005*,*Ruminococcaceae_NK4A214_gr oup*, *Alistipes*, *Lactobacillus*, *Oscillibacter*, *Alloprevotella*, *Parasutterella*, *Christensenellaceae_R-7_group*, and *Corynebacterium_1* were greater in response to BRS intake (Figure 3B and Table 4). In particular, the relative abundance of some bacteria was reversed to close to that of normal rats by BRS and orlistat (Figures 3C–E). *Turicibacter* in MC (2.47 ± 1.09%) and NC (0.72 ± 0.03%) decreased in LD (0.60 ± 0.12%), MD (0.91 ± 0.11%), HD (0.67 ± 0.17%), and PC (1.02 ± 0.15%). *Romboutsia* in MC (5.06 ± 1.97%) and NC (1.89 ± 0.08%) decreased in LD (2.27 ± 0.36%), MD (2.44 ± 0.29%), HD (1.87 ± 0.44%), and PC (3.10 ± 0.31%). *Oligella* in MC (1.51 ± 0.39%) and NC (0.31 ± 0.07%) decreased in LD (0.18 ± 0.08%), MD (0.33 ± 0.21%), HD (0.68 ± 0.47%), and PC (0.52 ± 0.36%), which fell sharply with BRS and orlistat treatment (*p* < 0.01). Therefore, BRS intervention increased the abundance of beneficial bacteria, such as *Cyanobacteria*, *Alistipes*, *Parabacteroides*, *Bacteroides*, *Ruminococcaceae*, *Lachnospiraceae*, and *Akkermansia*. At the same time, it inhibited the growth of bacteria including *Deferribacteres*, *Tenericutes*, *Turicibacter*, *Romboutsia*, and *Oligella*.

**TABLE 4:** Effects of BRS on gut bacteria at the genus level.

**Genus name**	**NC (%)**	**MC (%)**	**LD (%)**	**MD (%)**	**HD (%)**	**PC (%)**
*Bacteroides*	3.990.94	3.881.24	↑18.659.43^**##^	4.351.64	↑10.922.36^*#^	3.710.90
*Ruminococcaceae_UCG-014*	1.000.21	0.870.12	↑1.390.56^#^	1.100.17	↓0.490.09*	1.280.21
*Ruminococcaceae_UCG-005*	0.400.08	0.400.06	↑1.530.68^**##^	0.420.11	0.330.07	0.480.12
*Alistipes*	1.140.18	0.950.17	↑1.710.25^*##^	1.040.29	0.870.16	1.160.16
*Akkermansia*	0.690.13	0.630.43	↑1.290.16^*#^	0.330.38	0.640.09	0.380.26
*Parabacteroides*	0.390.13	0.250.07	↑0.880.36^*##^	0.290.13	↑0.690.34^#^	0.310.15

The values were expressed as mean ± standard error (SD). Statistical analysis was performed using a one-way analysis of variance (ANOVA) followed by LSD *post hoc* test. *p < 0.05 and **p < 0.01 vs. NC; #*p* < 0.05 and ##*p* < 0.01 vs. MC.

### BRS Modulated the Key Phylotypes of Gut Microbiota

**FIGURE 4:**
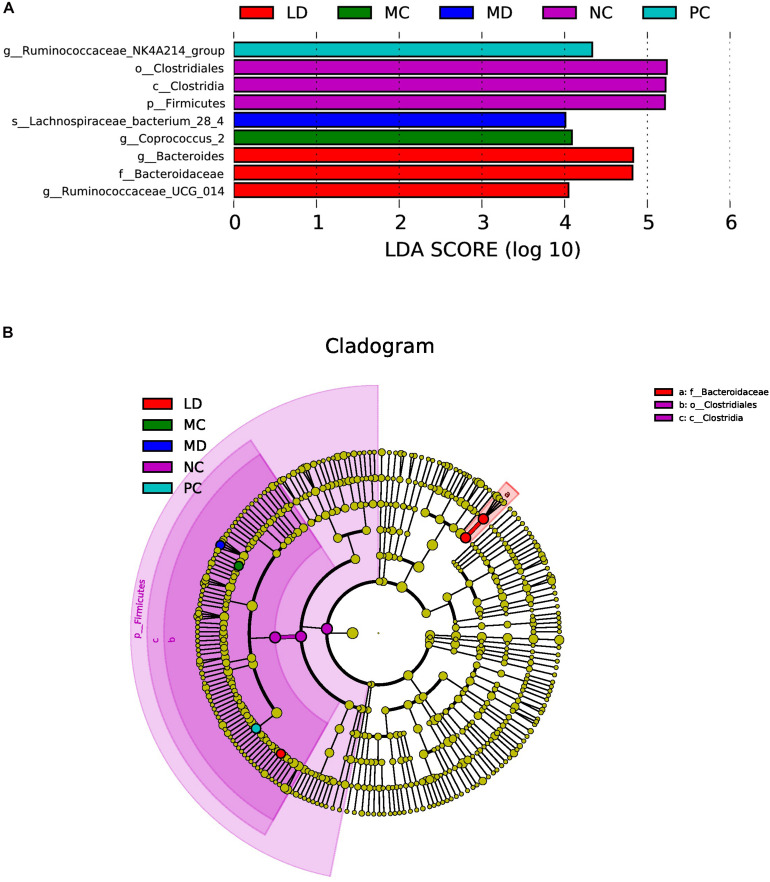
LEfSe analysis of gut bacteria in each group. **(A)** Linear discriminant analysis (LDA) score plot. Species with an LDA Score threshold >4 were listed and the length of the bar chart represented the impact of different species. **(B)** Taxonomy cladogram. The circle of radiation from inside to outside represented the taxonomic rank from phylum to genus (or species) and the diameter of the circles was based on relative abundance.

LEfSe was able to search for biomarkers with statistically significant differences from group to group and was applied to determine characteristic bacteria in each group. The results revealed that the NC group was rich in *Clostridiales*; in contrast, the MC group was characterized by a greater increase in the abundance of *Coprococcus_2*. After gavage of BRS, the intestinal flora of the LD group was markedly enhanced in *Bacteroides* and *Ruminococcaceae_UCG-014*, while the MD group was rich in *Lachnospiraceae_bacterium_28-4.* Orlistat treatment also notably increased the abundance of *Ruminococcaceae_NK4A214_group*. However, there was no significant change in the HD group, and only the NC group was detected to have a lower abundance of *Firmicutes* at the phylum level (Figures 4A,B). The results showed that BRS administration modulated the key phylotypes of gut microbiota by elevating the *Bacteroides*, *Ruminococcaceae_UCG-014*, and *Lachnospiraceae_bacterium_28-4* levels.

### Association Between Obesity-Related Biological Parameters and Gut Microbiota

**FIGURE 5:**
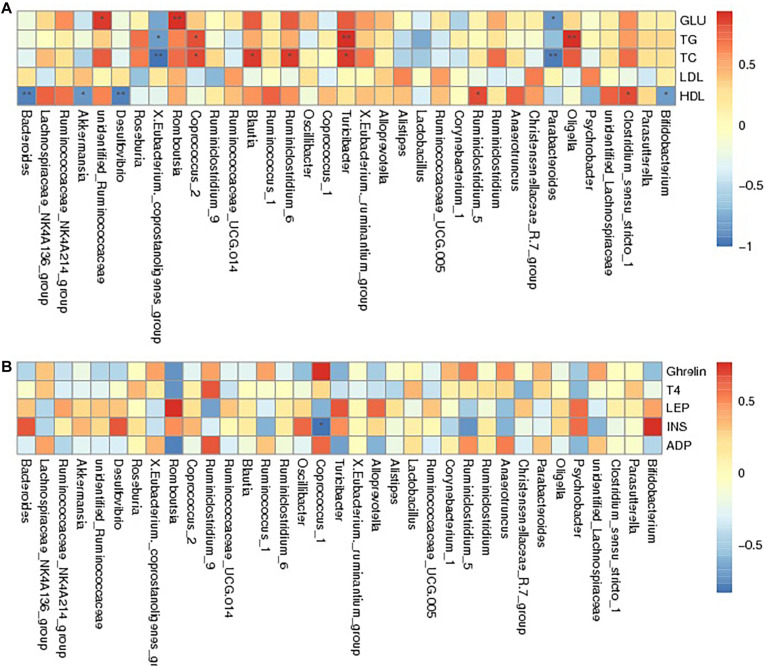
Spearman association analysis between gut microbiota and metabolic parameters at the genus level. The depth of the color corresponded the extent of relevance between gut microbiota and metabolic parameters, red meant positive correlation and blue meant negative correlation. **(A)** Glucolipid metabolism parameters and **(B)** Serum hormone. *Correlation was significant at the 0.05 level, **Correlation was significant at the 0.01 level.

Spearman correlation analysis was applied to assess the relationship between intestinal flora and the obesity-related biochemical indexes to identify whether there was a correlation between gut microbiota and host metabolism. The results showed that *unidentified_Ruminococcaceae*, *Romboutsia*, and *Turicibacte* were positively associated with the levels of GLU, TG, TC, LDL and HDL. *Bacteroides*, *Akkermansia*, *Desulfovibrio*, *X.Eubacterium._coprostanoligenes_group*, and *Parabacteroides* were negatively associated with GLU, TG, TC, and HDL levels and positively correlated with LDL levels. In contrast, bacteria including *Blautia*, *Ruminiclostridium_5*, *Clostridium_sensu_stricto_1*, and *Ruminiclostridium_6* appeared to have a positive relationship with GLU, TG, TC, and HDL levels and had a negative correlation with LDL levels. *Oligella*, *Coprococcus_2* and *Bifidobacterium* were positively related to GLU, TG, and TC levels, while they were negatively related to HDL levels (Figure 5A and Supplementary Table 1). The relationship between serum hormones and intestinal flora was studied, as shown in Figure 5B and Supplementary Table 2. *Romboutsia*, *Turicibacter*, and *Oligella* presented a negative relationship with ghrelin, ADP, and T4 and displayed a positive relationship with LEP and INS. However, *X.Eubacterium._coprostanoligenes_group*, *Coprococcus_1*, and *Anaerotruncus* were positively correlated with ghrelin, ADP, and T4 and negatively correlated with LEP and INS. Additionally, *Roseburia* was found to have a positive relationship with T4, ADP, and INS and to have a negative relationship with ghrelin and LEP. In summary, the change in gut microbiota regulated by BRS was linked with obesity-related blood indicators, suggesting that the BRS-induced weight loss may partly root in the impact on intestinal flora.

## Discussion

Our former researches showed that BRS could play a role in the prevention and control of obesity, and the dietary supplement of BRS could reduce body fat and weight (Wang et al., 2019). Increasing evidence showed that the levels of serum parameters and the relative abundance of gut microbiota will change dramatically with the development of obesity. However, the relationship between biological parameters and gut microbiota, and the anti-obesity mechanism of BRS have not yet been investigated. This study showed the effects of BRS on glucose and lipid metabolism, gut bacteria and their correlation, in order to understand the anti-obesity mechanism of BRS. Therefore, this study could provide the evidence and data for banana functional food development.

In this experiment, the concentrations of GLU, TG and TC were notably increased in high-fat diet-induced obese rats, and studies have demonstrated that the accumulation of serum lipids is considered to be the major factor in the risk of cardiovascular disease accompanied by an increase in GLU, TG, TC, and LDL-C levels and a decrease in HDL-C levels (Dyrbus et al., 2018). BRS significantly reduced GLU, TC, TG, and LDL-C levels in serum compared to the MC group during 6 weeks of BRS supplementation, which is consistent with previous literature (Lee et al., 2018), suggesting that BRS may play an effective role in ameliorating abnormal blood glucose and lipid metabolism induced by obesity.

Obesity is associated with changes in hormones that can send signals to the brain to modulate energy balance, including reducing energy consumption and increasing energy intake (Hariri and Thibault, 2010). High levels of insulin are a sign of insulin resistance caused by obesity; an abundance of adipose tissue reduces the sensitivity of cells to insulin, and islet cells are then stimulated to produce more insulin, resulting in hyperinsulinaemia. Generally, insulin resistance will elevate the level of free fatty acids (FFAs), decrease the transportation of glucose and then store it as glycogen, resulting in damaged glucose tolerance and an increase in blood glucose levels (Chen et al., 2018). As expected, the rats in the MC group had a significantly higher concentration of INS than those in the NC group, revealing that obese rats had severe insulin resistance. The research also showed that BRS could reduce INS significantly, which is consistent with previous studies (Maki et al., 2012). Meanwhile, with the increase in BRS dose, the insulin concentration gradually decreased in serum. Similarly, other hormones, including LEP, ADP, T4, and ghrelin, were also detected. LEP and ghrelin were associated with appetite and host energy balance. Similar to insulin resistance, there was resistance to leptin in obesity caused by excessive leptin content in serum and impaired energy homeostasis, leading to increased food intake and weight gain (Scarpace et al., 2005). In addition, the level of ghrelin was downregulated in obesity, and previous results indicated that ghrelin resistance may exist in obesity (Luna-Moreno et al., 2017). A low level of adiponectin in serum was related to chronic inflammation, and increasing adiponectin levels was conducive to preventing the occurrence of cardiovascular diseases through anti-inflammatory effects (Ohashi et al., 2010). According to the results, BRS treatment markedly decreased serum LEP levels and increased ADP, T4 and ghrelin levels compared with the MC group, which is consistent with previous research (Robertson et al., 2005; Shen et al., 2014). The data implied that BRS had potent effects on improving obesity-related hormone levels; furthermore, BRS could not only enhance the absorption and utilization of glucose and inhibit the rise in fasting blood glucose but also suppress the excessive secretion of insulin and improve the insulin sensitivity of cells.

The intestinal flora has a crucial influence on the human body, affecting the health and physiological functions of the host (Nicholson et al., 2012). Abundant evidence has indicated that gut microflora could be altered, including composition and diversity, in response to obesity caused by a high-fat diet (Damms-Machado et al., 2015; Liu et al., 2017). When the microbiome is distorted, dysbiosis may result in a disease state either by an excessive inflammatory response or poor immune system (Lumeng and Saltiel, 2011). Currently, diet is regarded as the key modulator in regulating disorders of the gut microbiota (Sonnenburg and Backhed, 2016). Therefore, long-term dietary intervention could be a potential, safe, and effective approach in the prevention and treatment of obesity. In the present report, alpha diversity was significantly enhanced by BRS treatment, especially in the MD group compared to the MC group, and the overall gut microbiota structure was distinctly shifted, as evidenced by PCA.

At the phylum level, all groups had the same species composition but different relative abundances. *Bacteroidetes* and *Firmicutes*, to our knowledge, co-exist in the human gut. The obese gut showed a tendency to reduce *Bacteroidetes* levels and increase *Firmicutes* levels, which was associated with host pathology (Ley et al., 2006). There was an outstanding decrease in the levels of *Bacteroidetes* in the MC group, and BRS could increase the *Bacteroidetes* levels and the ratios of B/F, which is consistent with a previous report (Parnell and Reimer, 2012). It is worth noting that in the MC group, the relative abundances of *Deferribacteres* and *Tenericutes* were sharply increased, while the relative abundance of *Cyanobacteria* was decreased. Although the difference was not significant in comparison with the NC group, the condition was significantly reversed after BRS treatment, which insinuated that BRS could improve gut bacterial structure. The above three bacteria belong to low-abundance bacteria (relative abundance < 1%), while the dominant bacteria provide an overview of healthy or diseased states. Some key organisms with low abundance are also essential (Benjamino et al., 2018). Recent studies have shown that *Cyanobacteria* have anti-inflammatory effects due to heightened IL-10 levels, and exogenous *Cyanobacteria* supplementation could retard blood glucose levels and lipid peroxidation (Pandurangan and Kim, 2016; Li et al., 2019). The enrichment of the *Deferribacteres* and *Tenericutes* population is a common phenomenon in obesity, which is positively linked with the pro-inflammatory factors IL-6, TNF-α, and IL-17A, causing aggravation of inflammation in obesity (Wang et al., 2017; Li et al., 2019). BRS may have a protective effect on the integrity of the intestinal barrier by upregulating the B/F ratio, inhibiting the overgrowth of inflammation-related bacteria (*Deferribacteres* and *Tenericutes*) and repairing adverse changes in intestinal flora caused by a high-fat diet.

At the genus level, BRS administration enriched the relative abundance of *Alistipes*, *Parabacteroides*, and *Akkermansia* and decreased the levels of *Turicibacter*, *Romboutsia*, and *Oligella*, which were dramatically increased in the MC group. The increasing proportion of *Turicibacter* was illustrated to have a side effect on lipid metabolism, which was positively correlated with TG, TC, and LDL-C levels and negatively correlated with HDL-C levels (Wan et al., 2018). *Romboutsia*, the characteristic microbes in HFD-fed rats, exhibited a positive relationship with indicators of body weight (waistline and BMI) and lipid levels (TG, TC, and LDL-C) (Zeng et al., 2019). The current research on *Oligella* mainly focused on the urinary tract, but it could be isolated from wounds, making it an opportunistic pathogen (Demir and Celenk, 2014; Wang et al., 2018). *Alistipes* played a vital role in the improvement in obesity-related clinical indicators, including body weight, blood pressure, glucose homoeostasis, and uric acid (Wan et al., 2018). Antagonistic substances produced by *Parabacteroides* could defend against the colonization of pathogenic bacteria and prevent the development of infectious diseases (Nakano et al., 2013). *Akkermansia*, a mucin-degrading bacterium, belongs to the *Verrucomicrobia* phylum, which has been shown to play an important role in maintaining a healthy mucus layer in the human gut by degrading mucus to produce oligosaccharides and SCFAs (Belzer and de Vos, 2012). On the other hand, the higher species abundance of *Akkermansia* trended toward a healthier metabolic status in overweight and obese people, alleviating the progression of obesity (Dao et al., 2016). Increased levels of *Alistipes*, *Parabacteroides*, and *Akkermansia* and decreased levels of *Turicibacter*, *Romboutsia*, and *Oligella* were considered to have beneficial potential in the healthy state of the human intestine. The results showed that BRS had the ability to regulate gut microbial disorders and improve the host’s metabolic function.

It was essential to confirm the characteristic bacteria in different groups, finding the key phylotypes of gut bacteria modulated by BRS. *Bacteroides* facilitated the degradation of various complex carbohydrates, such as glycans and starch, and generated SCFAs, such as acetic acid, propionic acid, and succinic acid (Roberfroid et al., 2010). In addition, the commensal factor (polysaccharide A) originating from *Bacteroides* could promote host immune function (Telesford et al., 2015). *Ruminococcaceae* was abundant in the large bowel and cecum of animals and humans and enriched in response to a high-RS diet (Abell et al., 2008). Numerous studies have demonstrated that *Ruminococcaceae* contribute to the degradation and fermentation of carbohydrates, favoring the production of SCFAs (Hooda et al., 2012; Shang et al., 2016). *Lachnospiraceae*, a butyrate-producing taxonomic core in healthy colons, dominated most individuals and synthesized butyrate through the acetyl-coenzyme A (CoA) pathway, which was supported by a meta-genomic data analysis (Vital et al., 2014).

It is widely believed that SCFAs have a beneficial effect on maintaining the health of colon cells and providing energy for the body (Topping and Clifton, 2001; Brahe et al., 2013). The abundance of *Bacteroides*, *Ruminococcaceae*, and *Lachnospiraceae*, as members of SCFA producers, was elevated in response to BRS. Based on this, a supposition was put forward that BRS was primarily fermented by *Bacteroides*, *Ruminococcaceae*, and *Lachnospiraceae* and subsequently produced SCFAs, which then had beneficial effects on its growth and colonization. It can be seen that obvious shifts in the populations of bacteria in the intestinal tract, on the one hand, inhibit the proliferation of *Turicibacter*, *Romboutsia*, and *Oligella*, and on the other hand, promote the growth of *Alistipes*, *Parabacteroides*, *Akkermansia*, *Bacteroides*, *Ruminococcaceae*, and *Lachnospiraceae*. In summary, BRS intervention may repair the imbalance of intestinal flora and be responsible for protecting the gut steady state.

This research provided further evidence that the gut microbiome participates in a variety of host metabolic processes, especially lipid and hormone levels, in obese rats. Combined with the shifted gut microbiota profile, a speculation was proposed that the healthy glycolipid metabolism cycle and hormone homeostasis were linked to the upregulation of beneficial bacteria and downregulation of harmful bacteria. Spearman correlation analysis confirmed that *Turicibacter*, *Romboutsia*, and *Oligella* (downregulated by BRS) were positively related to GLU, TG, TC, LEP, and INS and negatively related to ghrelin and ADP; *Bacteroides*, *Akkermansia*, and *Parabacteroides* (upregulated by BRS) were negatively related to GLU, TG, and TC. Generally, these key metabolites hold the potential to forecast obesity-related disease, and improvement based on these biomarkers might be beneficial to weight control and reduce the risk of dysmetabolism. Therefore, it is surmised that BRS could ameliorate host metabolism and relieve obesity by altering gut microbiota structure.

## Conclusion

(1) BRS reversed dyslipidaemia, controlled blood glucose stability, improved insulin sensitivity, and maintained hormone homeostasis in HFD-induced rats; in particular, BRS dose and hormone level showed a dose-effect relationship.
(2) BRS improved the diversity of gut microbiota and was responsible for the transformation in the overall structure of gut microbes, leading to a higher ratio of *Bacteroidetes*/*Firmicutes*; lower population of *Deferribacteres*, *Tenericutes*, *Turicibacter*, *Romboutsia*, and *Oligella*; and higher population of *Cyanobacteria*, *Alistipes*, *Parabacteroides*, *Bacteroides*, *Ruminococcaceae*, *Lachnospiraceae*, and *Akkermansia*.
(3) The change in gut microbiota induced by BRS was linked with obesity-related indicators (serum lipid, blood glucose, and hormone levels). *Turicibacter*, *Romboutsia*, and *Oligella* were positively related to GLU, TG, TC, LEP, and INS, while *Bacteroides*, *Akkermansia*, and *Parabacteroides* were negatively related to GLU, TG, and TC.
(4) The mechanism of BRS against obesity may be attributed to the manipulation of the intestinal microbiota that then improves glucolipid metabolism and guarantees hormone homeostasis, leading to a promotion in the state of host health and alleviation of obesity.

## Data Availability Statement

The datasets presented in this study can be found in online repositories. The names of the repository/repositories and accession number(s) can be found below: NCBI Sequence Read Archive accession numbers SRR13077928-SRR13077945.

## Ethics Statement

The animal study was reviewed and approved by the Experimental Animal Ethics Review Committee of South China Agricultural University, and the approval number was 2017-B13. The ethical care and use of laboratory animals followed the guidelines for Animal Experimentation in the animal research laboratories.

## Author Contributions

JF and YW made an equal contribution to this manuscript. JF analyzed and interpreted the data and contributed to writing the manuscript. YW contributed to the investigation and data curation. ST contributed to the investigation. JW contributed to the project administration, funding acquisition, writing—reviewing, and supervision. All authors contributed to the article and approved the submitted version.

## Conflict of Interest

The authors declare that the research was conducted in the absence of any commercial or financial relationships that could be construed as a potential conflict of interest.

## Footnotes

1 http://www.drive5.com/usearch/manual/chimera_formation.html

2 http://drive5.com/uparse/

3 http://drive5.com/uchime/uchime_download.html
