# Supplementary material for: Effects of Banana Resistant Starch on the Biochemical Indexes and Intestinal Flora of Obese Rats Induced by a High-Fat Diet and Their Correlation Analysis
Source: Front Bioeng Biotechnol. 2021 Jan 27;9:575724. doi: 10.3389/fbioe.2021.575724 (PMC7873301; doi:10.3389/fbioe.2021.575724)
Supplement: Supplementary file 1 [file Data_Sheet_1.docx]

# 1 Supplementary Figures and Tables

## Supplementary Tables

**Supplementary Table S1** Spearman association analysis between gut microbiota and glucolipid metabolism parameters at the genus level

| Bacterial genera |  | Glucolipid metabolism parameters | | | | |
| --- | --- | --- | --- | --- | --- | --- |
|  |  | GLU | TG | TC | LDL | HDL |
| *Bacteroides* | r | -0.200 | -0.203 | -0.314 | 0.232 | -0.928** |
|  | p | 0.704 | 0.700 | 0.544 | 0.658 | 0.008 |
| *Akkermansia* | r | -0.429 | -0.174 | -0.543 | 0.406 | -0.841* |
|  | p | 0.397 | 0.742 | 0.266 | 0.425 | 0.036 |
| *unidentified_Ruminococcaceae* | r | 0.886* | 0.348 | 0.771 | 0.058 | 0.638 |
|  | p | 0.019 | 0.499 | 0.072 | 0.913 | 0.173 |
| *Desulfovibrio* | r | -0.200 | -0.203 | -0.314 | 0.232 | -0.928** |
|  | p | 0.704 | 0.700 | 0.544 | 0.658 | 0.008 |
| *X.Eubacterium._coprostanoligenes_group* | r | -0.771 | -0.812* | -1.000** | 0.203 | -0.290 |
|  | p | 0.072 | 0.049 | 0.000 | 0.700 | 0.577 |
| *Romboutsia* | r | 0.943** | 0.638 | 0.714 | 0.464 | 0.493 |
|  | p | 0.005 | 0.173 | 0.111 | 0.354 | 0.321 |
| *Coprococcus_2* | r | 0.371 | 0.812* | 0.829* | -0.406 | -0.203 |
|  | p | 0.468 | 0.049 | 0.042 | 0.425 | 0.700 |
| *Blautia* | r | 0.771 | 0.493 | 0.886* | -0.232 | 0.580 |
|  | p | 0.072 | 0.321 | 0.019 | 0.658 | 0.228 |
| *Ruminiclostridium_6* | r | 0.771 | 0.493 | 0.886* | -0.232 | 0.580 |
|  | p | 0.072 | 0.321 | 0.019 | 0.658 | 0.228 |
| *Turicibacter* | r | 0.771 | 0.928** | 0.886* | 0.029 | 0.232 |
|  | p | 0.072 | 0.008 | 0.019 | 0.957 | 0.658 |
| *Ruminiclostridium_5* | r | 0.086 | 0.000 | 0.200 | -0.232 | 0.841* |
|  | p | 0.872 | 1.000 | 0.704 | 0.658 | 0.036 |
| *Parabacteroides* | r | -0.829* | -0.667 | -0.943** | 0.058 | -0.493 |
|  | p | 0.042 | 0.148 | 0.005 | 0.913 | 0.321 |
| *Oligella* | r | 0.314 | 0.928** | 0.771 | -0.319 | -0.116 |
|  | p | 0.544 | 0.008 | 0.072 | 0.538 | 0.827 |
| *Clostridium_sensu_stricto_1* | r | 0.600 | 0.551 | 0.714 | -0.232 | 0.841* |
|  | p | 0.208 | 0.257 | 0.111 | 0.658 | 0.036 |
| *Bifidobacterium* | r | 0.143 | 0.319 | 0.257 | 0.116 | -0.812* |
|  | p | 0.787 | 0.538 | 0.623 | 0.827 | 0.049 |

r: The r value meant correlation coefficient between -1 and 1, r<0 was negative correlation, r> was positive correlation.

p: p-value

*Correlation was significant at the 0.05 level, **Correlation was significant at the 0.01 level.

**Supplementary Table S2** Spearman association analysis between gut microbiota and serum hormones at the genus level

| Bacterial genera |  | Serum hormone | | | | |
| --- | --- | --- | --- | --- | --- | --- |
|  |  | Ghrelin | T4 | LEP | INS | ADP |
| *X.Eubacterium._coprostanoligenes_group* | r | 0.429 | 0.143 | -0.371 | -0.371 | 0.371 |
|  | p | 0.397 | 0.787 | 0.468 | 0.468 | 0.468 |
| *Romboutsia* | r | -0.714 | -0.714 | 0.771 | 0.486 | -0.771 |
|  | p | 0.111 | 0.111 | 0.072 | 0.329 | 0.072 |
| *Roseburia* | r | -0.029 | 0.371 | -0.029 | 0.200 | 0.029 |
|  | p | 0.957 | 0.468 | 0.957 | 0.704 | 0.957 |
| *Turicibacter* | r | -0.600 | -0.371 | 0.657 | 0.486 | -0.657 |
|  | p | 0.208 | 0.468 | 0.156 | 0.329 | 0.156 |
| *Lactobacillus* | r | 0.029 | 0.371 | -0.200 | 0.086 | 0.200 |
|  | p | 0.957 | 0.468 | 0.704 | 0.872 | 0.704 |
| *Oligella* | r | -0.200 | -0.029 | 0.257 | 0.257 | -0.257 |
|  | p | 0.704 | 0.957 | 0.623 | 0.623 | 0.623 |
| *Anaerotruncus* | r | 0.486 | 0.257 | -0.543 | -0.600 | 0.543 |
|  | p | 0.329 | 0.623 | 0.266 | 0.208 | 0.266 |
| *Coprococcus_1* | r | 0.771 | 0.257 | -0.600 | -0.829* | 0.600 |
|  | p | 0.072 | 0.623 | 0.208 | 0.042 | 0.208 |

r: The r value meant correlation coefficient between -1 and 1, r<0 was negative correlation, r> was positive correlation.

p: p-value

*Correlation was significant at the 0.05 level, **Correlation was significant at the 0.01 level.
